# Multimodal Imaging of Human Brain Activity: Rational, Biophysical Aspects and Modes of Integration

**DOI:** 10.1155/2009/813607

**Published:** 2009-06-15

**Authors:** Katarzyna Blinowska, Gernot Müller-Putz, Vera Kaiser, Laura Astolfi, Katrien Vanderperren, Sabine Van Huffel, Louis Lemieux

**Affiliations:** ^1^Department of Biomedical Physics, Warsaw University, Hoza 69, 00-681 Warszawa, Poland; ^2^Laboratory of Brain-Computer Interfaces, Institute for Knowledge Discovery, Graz University of Technology, 8010 Graz, Austria; ^3^Department of Computer Science and Systems, University of Rome “Sapienza”, Via Ariosto 25, 00185 Rome, Italy; ^4^Fondazione S. Lucia, Via Ardeatina 306, 00179 Rome, Italy; ^5^Research Division SCD, Department of Electrical Engineering, Katholieke Universiteit Leuven, 3001 Leuven, Belgium; ^6^Department of Clinical and Experimental Epilepsy, UCL Institute of Neurology, Queen Square, London WC1N 3BG, UK

## Abstract

Until relatively recently the vast majority of imaging and electrophysiological studies of human brain activity have relied on single-modality measurements usually correlated with readily observable or experimentally modified behavioural or brain state patterns. Multi-modal imaging is the concept of bringing together observations or measurements from different instruments. We discuss the aims of multi-modal imaging and the ways in which it can be accomplished using representative applications. Given the importance of haemodynamic and electrophysiological signals in current multi-modal imaging applications, we also review some of the basic physiology relevant to understanding their relationship.

## 1. Introduction

Since the mid 1990s there has been an increase in interest in synchronous multi-modal imaging, whereby two modalities or more are used simultaneously, which has arisen in large part from investigators interested in the study of spontaneous brain activity and in particular epilepsy. Although the electroencephalogram (EEG) was previously combined with PET, the advent of simultaneous EEG-fMRI (and optical imaging techniques, such as NIRS) with temporal resolutions of the order a second or less has lead to multiple applications in and outside the field of epilepsy.

The overarching motivation for integrating data from multiple modalities is to gain a more complete picture of the brain activity of interest. Implicit in the multi-modal integration or multi-modal imaging is the notion that all measurements relate to the same activity in space and time. Therefore at the most basic level, multi-modal integration can mean spatial coregistration of the observations. Coregistration in time can mean two things: either measurements are performed simultaneously (same time of day), simultaneous EEG-fMRI of randomly occurring epileptic discharges being an example; or monomodality measurements made at the same time relative to an event but not simultaneously, that is, serially. Examples include separate ERP and fMRI studies in relation to the same stimulus subsequently brought together through correlation of the responses (as a function of some externally controlled factor) or the spatial coregistration of independently derived source localisation estimates. Serial multi-modal integration implies a degree of predictability and more importantly reproducibility of the events: the retrospective integration of serially acquired datasets is actually restricted to the reproducible aspects of the activity of interest, such as effects averaged across repeated events.

The integration of electrophysiological and haemodynamic signals (BOLD, CBF, CBV) is particularly important in the context of this discussion for two reasons: their intrinsic importance and complementarity, and data availability. Electrophysiological signals are particularly important given the direct link between EEG/MEG and neuronal synchrony. On the other hand localisation based on EEG/MEG is fundamentally limited. The more indirect link between haemodynamic signals and neuronal activity is partly compensated by our capacity to obtain 3D maps covering almost the entire brain and with good spatial resolution particularly for fMRI. While numerous observations have shown that BOLD changes can be related with various forms of brain activity, such as the haemodynamic response to external stimuli recently, which makes fMRI possible and useful, much remains to be learnt. Recently, experiments have focused more specifically on the relationship between neuronal activity measured at the microscopic level and the BOLD effect.

## 2. Basic Studies of the Relationship between the BOLD Signal and Brain Activity

### 2.1. The Neuronal Correlates of the BOLD Signal

**Figure 1:**
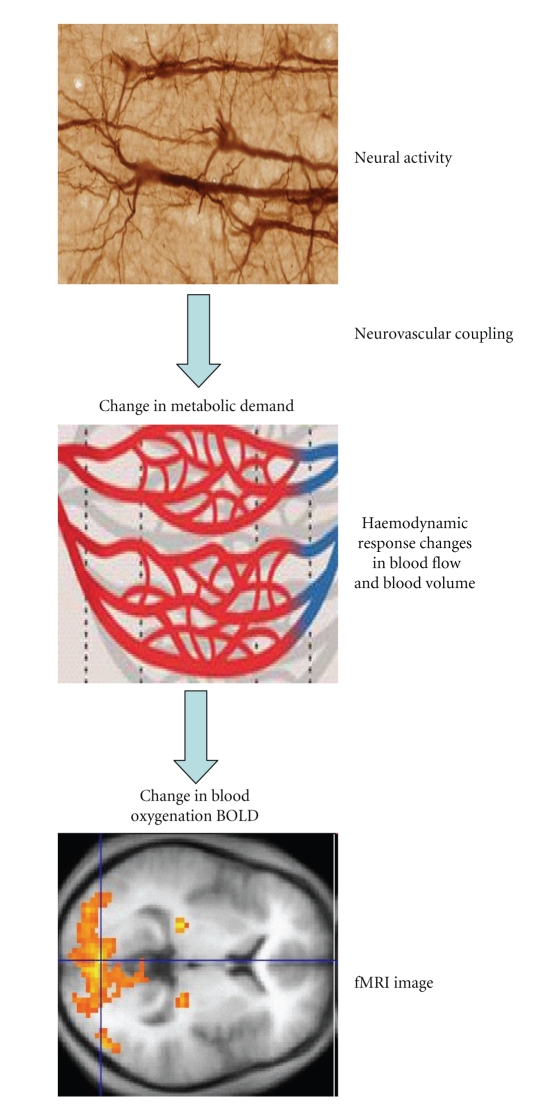
From neural activity to fMRI image.

The chain of events and factors that links neuronal activity to BOLD signal change is long (Figure 1), and the transitions between them are far from simple. Neural activity through neurovascular coupling influences the metabolic demand. Metabolic changes impact on haemodynamic response which is dependent on physiological factors such as local cerebral blood flow, deoxyhaemoglobin/oxyhaemoglobin ratio, blood volume, and vascular geometry. Therefore, inferences in fMRI concerning neural activity rely on the accuracy, validity, and efficiency of prespecified models and hypotheses.

A model elucidating the basis of BOLD signal was formulated by Friston et al. [1], furthering the Balloon model of Buxton et al. [2], which described how evoked changes in blood flow were transformed into fluctuations in blood oxygenation level. The Balloon model was embedded in a haemodynamic input-state-output model that included the dynamic of perfusion changes that are contingent on underlying synaptic activation. In the model of Friston et al. it was assumed that neural activity is linearly coupled to the metabolic demand, but the relationship between blood flows and BOLD is non-linear. The model provides a level of explanation for the biphasic shape of the haemodynamic response function, with a positive peak around 6 seconds following event or stimulus onset, followed by a negative undershoot at around 15 seconds (first to second peak amplitude ration ~6) and gradual recovery to baseline.

However, a general assumption is of a linear relationship between certain measures of neural activity and BOLD signal, which finds confirmation in some experimental studies for example: [3, 4]. Nevertheless it was demonstrated that nonlinear refractoriness of BOLD responses can occur at very short interstimulus intervals [5]. It was also reported in another study that BOLD signal increases linearly with positive stimulus amplitude, but for negative amplitude of stimulus highly nonlinear behaviour ensues and that the response to a second stimulus was compromised by first, evidencing a nonlinear refractoriness of the BOLD response, possibly of haemodynamic origin [6]. These studies point out that the results of the fMRI studies of evoked activity depend strongly on the choice of the interstimulus interval.

Several factors in the chain of events from neuronal activity to vascular changes are difficult to account for in models of the BOLD effect. For example, the details of the vascular architecture and the presence of large veins in the vicinity of the activated neurons. Microvascular density, which is lower than that of neurons and is affected by large vessel contribution, may influence the results and may be a limiting factor of spatial resolution of BOLD signal [7]. Which aspect, or expression, of neuronal activity is best reflected in the BOLD signal namely potential firing versus synaptic activity remains unclear. This problem was reviewed in [8, 9], where the contradictory opinions were discussed. Namely the empirical evidence was quoted suggesting that the spikes generated by cortical cells contribute little to the metabolic demand of brain, accounting for only 3*%* of the resting cortical energy consumption; also experiments performed on rates show that up to 95*%* of regional cerebral blood flow increases might be dependent on postsynaptic activity. However another contribution reported a correlation between spiking activity and BOLD signal [10]. In fact, spiking activity and synaptic potentials are related to each other. Nevertheless the comparative studies indicated that the BOLD signal matched (Local Field Potentials) LFPs better than multiunit spiking activity [4].The findings of the same study suggest that the BOLD contrast mechanism reflects the input and intracortical processing of a given area rather than its spiking output. 

BOLD decreases (sustained decreases, in contrast to the transient negative undershoot of “positive” haemodynamic response function) have been reported in relation to some stimuli and events, such as epileptic spikes. Simultaneous fMRI measurements and electrophysiological recordings revealed a negative BOLD response beyond the stimulated regions of visual cortex, associated with local decreases in neural activity as expressed in terms of LFP power below the level of spontaneous (background) activity [11].

The relationship between neuronal inhibition and BOLD is currently under debate. Since the inhibitory activity similarly to excitatory processes requires energy, inhibition can be associated with increased metabolic demand which may be reflected as BOLD increase. On the other hand most connections in the brain are excitatory and decrease of excitatory activity caused by inhibition may lead to a decrease of blood flow. Experimental results point out that both arguments may be valid. Mathiesen et al. [12] found comparable cerebral blood flow increases during stimulation of excitatory and inhibitory pathways in cerebellum. However, studies using agonists of inhibitory transmitters have generally shown decreases in measured energy metabolism for example: [13, 14]. Modelling studies [15] have demonstrated that there are several factors that may play the role in the impact of inhibition on imaging results: local connectivity, type of inhibitory connection, and the kind of task. Depending on these factors neuronal inhibition may result in BOLD increases if the region is not driven by excitation or there is low local excitatory recurrence. Alternatively for active excitation or high recurrence, inhibition may lead to BOLD decreases.

Another interesting and still unresolved problem is whether fMRI can differentiate between small activity changes in large cellular populations, and large changes in small populations. The resolution in typical fMRI scanner is ~8–50 mm^3^, which corresponds to at least 10^6^ neurons. The highest resolution corresponds to one cortical column which contains 10^5^ neurons. Therefore in the case of an apparatus of typical resolution several neural populations of different activity patterns may be scanned.

### 2.2. BOLD versus Brain Oscillations

At the macroscopic level, while BOLD reflects the number of active neurons, EEG/MEG amplitude depends primarily on the number of neurons acting synchronously. As was pointed out by Nunez [16] the activity of synchronously acting neurons is proportional to their number and for asynchronously acting neurons it is proportional to the square root of their number. There are about 10^8^ neurons located within range of a standard EEG electrode; supposedly all of them are continuously active, but only 1% of them acting synchronously. The latter's contribution to the scalp signal will be 10^6^/sqrt (10^8^–10^6^), that is, 100 times greater than the 99% nonsynchronised neurons. Therefore asynchronous neural activity will be hardly reflected in EEG, in contrast to fMRI. 

The synchronous action of neural populations gives rise to the characteristic EEG rhythms, which have specific roles in the information processing by brain. Specific tasks such as movements or perceptions are connected with the synchronization and desynchronization of EEG in specific frequency bands. It is therefore of importance to establish the relations between the electroencephalographic rhythmical activity and fMRI results. A heuristic model relating haemodynamic changes to the spectral profile of ongoing EEG activity was elaborated by Kilner et al. [17]. The assumptions of the model were that the BOLD signal is proportional to the rate of energy dissipation, where dissipation was expressed as a product of trans-membrane potential and current. The authors found that the metabolic response is proportional to the “effective connectivity” and temporal covariance of the trans-membrane potentials. “Effective connectivity” in the sense of synaptic efficacies was expressed as a Jacobian *J*, with diagonal elements reflecting effective membrane conductance. The measure of change of effective connectivity is expressed by parameter *α*, defined by,

(1) $$J\left(\alpha \right)=J\left(0\right)+\frac{\alpha \partial J}{\partial \alpha },$$

where *J*(*α*) = *J*(0) corresponds to the resting state. This model of activation postulates that an increase of *α* caused an acceleration of dynamics of the neural system and consequent increase of the system's energy dissipation. Next the authors connected the acceleration with the spectral properties of the system. Namely they have shown that the activation modulates the EEG spectral density *g*(*ω*) according to

(2) $$\tilde{g}\left(\omega \right)=\frac{g\left(\left(1+\alpha \right)\omega \right)}{1+\alpha },$$

where *ω* is the circular frequency and $\tilde{g}$ is the modulated spectral density.

From (1) and (2) it follows that activation causes a shift of EEG spectral profile towards higher frequencies with amplitude decrease. This means that as neuronal activation increases there is a concomitant increase in BOLD signal and shift in the spectral power towards higher frequencies.

Indeed, the relative decrease of BOLD signal for low frequency EEG rhythms and an increase in high frequencies found experimental confirmation. The simultaneous EEG/fMRI studies have shown that alpha rhythm is negatively correlated with BOLD signal, for example: [18, 19], also in the experiment involving low-frequency entrainment the reduction of BOLD was reported [20]. In the same publication predominantly positive correlations between EEG and fMRI were found in the higher frequency bands: 17–23 Hz and 24–30 Hz.

These findings support the model; however it does not account for some low frequency phenomena in brain, as the authors of [17] admit themselves. Namely it has been reported that very slow EEG activity fluctuations in the monkey visual cortex were reflected in the BOLD signal [21]. It seems that the further studies concerning simultaneous EEG-fMRI studies as well as improvement of models are needed to unravel the mechanisms underlying manifestation of rhythmic brain activity in the imaging studies.

Finally, the relationship between electrophysiological signals and BOLD will be greatly affected by the fluctuations of the background activity which may influence the evaluation of evoked activity hampering the estimation of stimulus-related responses. On the other hand the fMRI investigation of spontaneous activity offers the new possibilities in the investigations of brain rhythms, sleep patterns, and epilepsy [8]. 

## 3. Modes of Multimodal Fusion at the Macroscopic Level

### 3.1. EEG versus FMRI: Illustration in Motor Imagery

The purpose of even more advanced fusion of multi-modal data in relation to a specific type of brain activity is to overcome some of the limitations of individual measurements. In this section we will consider examples of single-modality studies of a specific cognitive, namely, motor imagery. More specifically, we will review and contrast investigations of motor execution, passive movements, and movement imagination in spinal cord injured (SCI) using EEG and fMRI separately.

In an EEG study by Müller-Putz et al. [22] event-related desynchronization/synchronization (ERD/ERS) patterns in paraplegic patients (suffering from a complete spinal cord injury) are compared with able-bodied controls during attempted (active) and passive foot movements. The aim was to address the question, whether patients do have the same focal beta ERD/ERS pattern during attempted foot movement as normal subjects. For this purpose EEG was recorded from sixteen sintered standard scalp electrodes. The results showed a mid-central focus of beta ERD/ERS patterns during passive, active, and imagined foot movements in normal subjects. This is in contrast to a diffuse and broad ERD/ERS pattern during attempted foot movements in patients. Only one patient showed an ERD/ERS pattern similar to able-bodied subjects. Furthermore, no significant ERD/ERS patterns during passive foot movement in the group of the paraplegics were found. In a further EEG study [23] a 3-class Brain-Computer Interface motor imagery screening (left hand, right hand, feet) was performed in a group of able-bodied and spinal cord injured participants. EEG was recorded from 15 scalp electrodes. Comparing Brain-Computer Interface classification accuracy we found a significantly lower classification rate in the patients compared to the healthy subjects. In conformity with the results discussed above, ERD/ERS patterns are diffuse and scattered in the patients group.

Using fMRI Alkadhi et al. [24] measured imagination of foot movements in able-bodied and SCI participants (lesion height from Th3 to L1, range of age was 22–43 years). They found that the degree of BOLD activation (contra-lateral M1 and S1 foot representation; bilaterally SMA, pre-SMA, CMA, and further) was significantly higher in the SCI patients as compared to the able-bodied participants. It is of interest to note that the SCI patients showed a strong correlation with their vividness scores for motor imagery. One explanation for the enhanced activation in SCI patients could be that they were engaged in the task and displayed a higher mental effort as compared to the able-bodied subjects. In a further fMRI study Enzinger et al. [25] compared BOLD patterns of motor imagery patterns in a remarkable SCI patient, who was well trained in motor imagery (extensive training for a period of several years), to a group of able-bodied controls. In the patient significant activation of sensorimotor networks (sensorimotor cortex contralateral to side of movement imagination, SMA, pre-SMA, and further) occurred during imagery of repetitive hand and foot movements (versus rest); whereas in able-bodied subjects significant activation only occurred in relation to hand motor imagery and only in premotor areas (pre-SMA). No significant activation could be demonstrated within the sensorimotor cortex in the control group. The pattern of activation found in the patient during motor imagery corresponded to the pattern of activation found in controls during motor execution.

The possible explanations for the contrasting results of the EEG and fMRI studies include: experimental factors (such as intersubject variability and differences in experimental conditions) or biological factors. Using simultaneous EEG and fMRI acquisitions would eliminate intersession (and therefore intermodality) experimental confounds opening the way for greater biological insights. For example, one could assess whether the results reflect a mismatch between the BOLD effect, and whichever aspect of the EEG was used. Such studies have already been performed for ERD. For example Formaggio et al. [26] used combined EEG-fMRI over motor areas during finger movements and found a negative correlation between EEG power changes and BOLD activity contra-lateral to the movement. Significant ERD in alpha and beta frequency bands were associated with activation of the anterior and posterior central sulcus in both sensorimotor areas.

### 3.2. FMRI-Informed EEG: Haemodynamic Priors for Cortical Activity and Conectivity Estimation 

In this section, we will review some examples of how information obtained from fMRI recordings can be used to improve the accuracy of the estimation of the cortical activity and connectivity from EEG recordings. There is experimental evidence suggesting that the estimation of the cortical activity performed with the use of neuroelectromagnetic recordings improves with the use of the haemodynamic information recording during the same task [27–30]. This was also demonstrated during simulation studies [27, 29, 31]. 

In [32] the impact of the use of a priori information from fMRI recordings in the EEG-based estimation of the cortical activity and connectivity was reported. The data used were related to high resolution EEG and fMRI signals collected during visually triggered finger tapping movements in four healthy subjects. The methods include the use of subjects' multicompartment head models (scalp, skull, dura mater, cortex) constructed from MRI, multidipole source model, and regularized linear inverse source estimates of cortical current density [33, 34]. The priors in the resolution of the linear inverse problem were derived from the haemodynamic responses of the cortical areas as revealed by block-designed (strength of activated voxels) and event-related (coupling of activated voxels) fMRI. The multimodal integration of EEG and fMRI data was performed using a metric which takes into account the haemodynamic information offered by the recorded fMRI data as a norm in the source space. As described in [31] the contribution of the fMRI priors in the estimation of the cortical current density is given by the statistically significant percentage increase of the fMRI signal during the task, compared to the rest state. The statistical significance of the cortical activity obtained was assessed by computing the z-score with respect to the rest period. Cortical activity was significantly increased in the left ROIs representing parietal (BA 5), premotor (BA 6A), sensorimotor (BA 3, 2, 1, BA 4), and prefrontal (BA 8 and BA 9) cortical areas, and similarly for the ROIs located in the right hemisphere in premotor (BA 6A) and prefrontal (BA 8) cortical areas. 

Connectivity estimations on the cortical waveforms obtained by the multimodal integration of EEG and fMRI recordings were performed. The connectivity was estimated by means of the Directed Transfer Function [35], a method to determine the directed influences between any given pair of signals in a multivariate data set. The approach is based on the concept of Granger causality and uses a multivariate autoregressive model (MVAR) simultaneously modelling the entire set of signals [36]. The application of DTF to linear inverse estimate of the cortical activity was described in [37–39].

The main results obtained with the multimodal integration of ERP and fMRI data were related to the activity of a network involving the right frontoparietal cortical structures. The flow of the connections moved from the parietal and premotor areas towards the right and left prefrontal ones. The ROIs located at the parietal (B.A.5 ) and premotor areas (B.As 6) revealed as the source of an activity that spreads and reaches virtually all the other ROIs considered, from the occipital (B.A. 19) to the prefrontal (B.A. 9, 46) areas of both hemispheres. 

These results were compared to those obtained on the same data set in [38] with the use of the EEG data without fMRI priors. A substantial agreement between the two sets of connectivity patterns (with and without fMRI priors) can be appreciated, although differences are present in some cortical areas, in the intensity of the cortical connections. While the parietal and frontal connections are revealed in both the estimations, a shift of the intensity is observed in the connectivity patterns computed by using EEG and fMRI information when compared to those obtained using only the EEG data.

 Similar results were obtained by estimating the cortical connectivity patterns in the beta band from the high resolution EEG recordings obtained during the execution of the Stroop task, with and without fMRI priors [40, 41]. In this case it can be noted that a substantial agreement exists for the connectivity patterns obtained, that show an involvement of the parietal and the frontal areas. This finding is similar to that already observed in the finger tapping experiment, as in this case the intensity of the DTF estimated by the cortical waveforms obtained with the multimodal integration was significantly higher than that obtained by using only the EEG information. Slight differences in the cortical pattern in different cortical areas were noted.

We conclude that the inclusion of fMRI priors in the estimation of cortical activity and connectivity from high resolution EEG can add information to the estimation and help to define the role of some specific cortical areas in the flow of information necessary to the execution of a specific task.

### 3.3. Simultaneous EEG-FMRI of Spontaneous Brain Activity: Epilepsy

Simultaneous multi-modal acquisitions make it possible to acquire signals in identical experimental conditions. Assuming that data quality is preserved continuous acquisition synchrony means that the same events are captured and can be studied across modalities. Therefore synchronous multi-modal acquisitions make it possible to study signals related to events over which one has no experimental control, that is, spontaneous brain activity in the resting state. An important example of this is epileptic activity which can be captured on EEG, an important investigative and clinical tool in the field of epilepsy (And visually of course, in the case of seizures; there have been a number of (single-modality) fMRI studies of seizures, for example, [42–44].).

Due to its potential clinical impact in cases with drug-resistant epilepsy considered for surgical resection, localisation of the generators of epileptic activity is a central issue in the field of epilepsy imaging. Although EEG provides important clinical information its ability to localise generators is fundamentally limited by the nonunicity of the inverse electromagnetic problem [45]. However, tomographic modalities such as fMRI do not suffer from this limitation. Therefore, as early as 1993, only a few years following the demonstration of BOLD fMRI, investigators in Boston started working on combined EEG and fMRI acquisitions [46–48]. The key driver for this methodological development is the lack of overt manifestation during or following an interictal spike (in contrast to seizures, which are rarer and generally more difficult to study using fMRI due to motion and safety considerations). In the context of fMRI this means that the only way of tagging scans according to brain state (e.g., IED versus. background) necessary for modelling the BOLD changes is to record the EEG simultaneously.

Studies using EEG-fMRI applied to epilepsy generally follow the asymmetric, EEG -> fMRI, mode of integration: the EEG's sole purpose is as a basis for fMRI modelling, that is, to answer the question, what are the BOLD correlates of the EEG events? For example, early applications used a spike-triggered acquisition mode whereby each fMRI scan (or burst of scans) was acquired following the visualisation of a spike, and a corresponding number of scans acquired following periods of background activity [49–54]. It is important to remark that this (nonperiodic) interleaved acquisition scheme was also motivated by one of the key issues in synchronous multi-modal data acquisition: data quality degradation due to interaction between each modality's hardware. In the case of EEG recording inside MR scanners, the problem of pulse-related and image acquisition artefacts arises. By implementing a pulse-artefact reduction scheme, the authors were able to increase the reliability of spike detection; by limiting scanning to short bursts following events of interest, they limited the impact of the image acquisition artefact [50, 51]. Subsequently, the development of software techniques to allow recording of good quality EEG during continuous scanning gave rise to the more flexible technique of continuous and simultaneous EEG-fMRI [55].

In the spike-triggered approach spike-related activation was determined by applying voxel-wise t-tests across the two scan sets. For datasets acquired using the analytical framework of event-related designs is employed, whereby EEG events of interest are identified, marked, and represented mathematically to form the basis of a general linear model of the entire BOLD time course and conforms to the EEG -> fMRI, mode of integration [55, 56]. In an extension of the straightforward EEG -> fMRI mode of integration Liston et al. tested the significance and localisation of BOLD changes linked to EEG epochs below the threshold of visual spike detection, but marked as possible spikes by an automated algorithm, and were able to demonstrate the epochs' probable epileptiform nature [57]. In the not so distant future, we envisage that the availability of biophysical models linking neuronal activity to EEG and BOLD signals that can be inverted should result in more symmetrical improved estimation of neuronal generators.

### 3.4. Simultaneous ERP-FMRI: Single Trials

Event-related potentials (ERPs) are EEG responses to specific sensory, cognitive and motor events [58]. Despite the rich temporal information provided by ERPs, they suffer from the same spatial resolution limitations as other scalp EEG patterns. The integration of ERP and fMRI may provide a more complete spatiotemporal characterisation of evoked responses through the study of individual trials.

To this end a major breakthrough was achieved when simultaneous EEG and fMRI recordings became feasible [59], safe [60], and of sufficient quality [61–63]. As mentioned above, synchronous acquisitions remove intermodality bias relative to experimental conditions [64]; for example, Novitski et al. [65] showed, that the loud noise of the scanner can influence how the brain reacts to certain stimuli. In addition, simultaneous recordings allow the investigation of relationships between event-related potentials and BOLD responses across individual events. 

In the following, we illustrate four approaches to the analysis of simultaneously acquired ERP-fMRI, namely, ERP-informed fMRI analysis, the use of constrained source localisation, parallel independent component analysis (ICA), and joint ICA.

The general idea behind *ERP-informed fMRI* analysis is to identify brain regions with fMRI responses that reflect paradigm-related amplitude modulations in individual ERPs. In [66], for instance, EEG data were acquired during an auditory target detection (oddball) paradigm and both ICA and wavelets were applied to denoise the data. Subsequently single-trial N1, P2 and P3 amplitudes [67] were extracted from the data. The resulting vectors were convolved with the haemodynamic response function (HRF) and used as regressors in a general linear model (GLM) for the BOLD time course. The findings confirmed that the combination of ERP and fMRI enables identification of regional responses in the fMRI, reflecting a specific aspect of varying potentials. Similarly, Debener et al. [68, 69] applied ICA to isolate task-related activity from a typical EEG mixture of overlapping brain and nonbrain sources. These single-trial amplitudes from selected independent components preserve event-related trial-by-trial fluctuations within each condition and can thus be correlated with the BOLD response. This research showed that ICA is a practical solution to minimise artefacts and identify functionally meaningful EEG activity on a trial-by-trial basis. Another example of this approach can be found in [63] where subjects performed an auditory oddball task during simultaneous EEG-fMRI measurements. They showed significant BOLD effects related to ERP latency and amplitude.

In a further refinement, regions of interest (ROIs) were extracted from the fMRI maps [70] and used as *constraints* in *source localisation* analysis. The cortical generators thus found, corresponded highly to findings by means of intracranial measurements and the different timing of activations associated with the task paradigm could also be appreciated. As such, this method improved the solution of the inverse EEG problem and enabled the study of the dynamic behaviour of ROIs. 

The abovementioned methods rely on the assumption that scalp EEG data from both a selected channel and latency can predict BOLD changes in single voxels [71]. However, this is not physiologically plausible, because event-related processes might be spatially and temporally mixed across the brain. Therefore, Eichele et al. [71] propose “*parallel ICA*” to unmix EEG and fMRI separately and to match temporal sources from the EEG with spatial sources from the fMRI.

The above approaches are asymmetric in the use of data, namely, EEG for analysis of fMRI data or vice versa. Moosmann et al. [72] propose a symmetric approach by not only combining EEG and fMRI in one common data space but also by applying a *joint ICA* model to these data. Therefore features of neural sources whose trial-to-trial dynamics are jointly reflected in both modalities can now be studied.

ERP-informed fMRI is so far the most widely validated method; it has already been studied in numerous applications. Since it allows tracking and correlating the trial-to-trial variability of both EEG and fMRI, it provides detailed information about regional fMRI responses with the temporal accuracy typical of EEG. Furthermore, the second proposed method, constrained source localisation, is based on the same principles but additionally uses the ERP-informed fMRI regions as constraints for further source localisation. Unfortunately, both these methods suffer from several important drawbacks. First of all they leave room for improvement concerning the proportion of EEG data used for integration [72]. The reason for this is that all these studies take into account only certain features of the data and therefore possibly disregard important temporal or spatial information. In addition, the observed data from both modalities often represent a mixture of signals coming from multiple neural sources. A voxel-by-voxel prediction of the fMRI signals based on the ERP data (even after application of ICA on these ERPs) may therefore become unreliable when multiple sources contribute to either the predictor or the response variables. The parallel ICA approach tries to address both these issues, but still shares a disadvantage with the above methods, namely, the asymmetry of the procedure. In the joint ICA approach both modalities are therefore assembled and decomposed in one common data space. As such an asymmetric information flow is no longer present. A limitation of joint ICA is however that it cannot reflect the time domain of event related oscillations that are not time locked within one component [72].

ICA is clearly emerging as an important analytical tool, reflecting the exploratory nature of work on EEG and fMRI data fusion at the level of single events. However, the relevance of the assumptions on which ICA is based with regard to the separation of the source activity into electrical and haemodynamic components requires further testing. Furthermore, not enough validation has yet been performed to conclude whether one of the above methods performs much better than the others. So far the performance of the methods seems highly dependent on the application of interest.

## 4. Conclusion

The relationship between the BOLD signal and neural activity is complex, and depending on the experimental paradigm it can be linear or nonlinear. The relative contributions of slow wave activity versus spiking activity and the dependence of BOLD on spectral properties of neural activity also remain to be fully characterised but increasing evidence indicates that Local Field Potentials and the higher frequency components of brain rhythms are good correlates of the metabolic response. Experimentally, various modes of multi-modal image integration are available to the investigator. The inclusion of fMRI priors in the linear inverse estimation of the cortical activity can be used to increase the spatial resolution of the EEG and improved estimation of cortical connectivity, which is one of the most challenging and important objectives of neuroimaging. Synchronous acquisitions represent the most flexible solution in terms of analysis, allowing full exploitation of the data at the available temporal and spatial resolution, down to individual events and free of intersession bias. In the field of motor imagery, this approach has demonstrated a negative correlation between event-related desynchronization and the BOLD signal. In the field of epilepsy, simultaneous EEG-fMRI is necessary for the study of the haemodynamic correlates of interictal pathological discharges due to their subclinical nature. Such studies have demonstrated BOLD increases and decreases in relation to sharp waves and sharp- and slow-wave complexes. In the field of evoked response studies we envisage the extension of the connectivity estimators in the time-frequency domain, which would return more detailed information about the functional links established between different cortical sites during the evolution of the task. The proposed fusion procedures for single trial ERPs and fMRI enable us to study the temporal dynamics the spatial behaviour of information processing and cognitive functioning in greater detail. Future developments in biophysical modelling will permit more precise and complete estimations of neuronal activity using noninvasive means. This may lead to more symmetric data analysis approaches better capable of identifying salient spatiotemporal patterns and assist in the design of efficient experimental strategies.

## References

[1] Friston KJ, Mechelli A, Turner R, Price CJ (2000). Nonlinear responses in fMRI: the balloon model, Volterra kernels, and other hemodynamics. *NeuroImage*.

[2] Buxton RB, Frank LR (1997). A model for the coupling between cerebral blood flow and oxygen metabolism during neural stimulation. *Journal of Cerebral Blood Flow and Metabolism*.

[3] Arthurs OJ, Williams EJ, Carpenter TA, Pickard JD, Boniface SJ (2000). Linear coupling between functional magnetic resonance imaging and evoked potential amplitude in human somatosensory cortex. *Neuroscience*.

[4] Logothetis NK, Pauls J, Augath M, Trinath T, Oeltermann A (2001). Neurophysiological investigation of the basis of the fMRI signal. *Nature*.

[5] Friston KJ, Josephs O, Rees G, Turner R (1998). Nonlinear event-related responses in fMRI. *Magnetic Resonance in Medicine*.

[6] Mechelli A, Price CJ, Friston KJ (2001). Nonlinear coupling between evoked rCBF and BOLD signals: a simulation study of hemodynamic responses. *NeuroImage*.

[7] Turner R (2002). How much codex can a vein drain? Downstream dilution of activation-related cerebral blood oxygenation changes. *NeuroImage*.

[8] Logothetis NK (2008). What we can do and what we cannot do with fMRI. *Nature*.

[9] Arthurs OJ, Boniface S (2002). How well do we understand the neural origins of the fMRI BOLD signal?. *Trends in Neurosciences*.

[10] Rees G, Friston K, Koch C (2000). A direct quantitative relationship between the functional properties of human and macaque V5. *Nature Neuroscience*.

[11] Shmuel A, Augath M, Oeltermann A, Logothetis NK (2006). Negative functional MRI response correlates with decreases in neuronal activity in monkey visual area V1. *Nature Neuroscience*.

[12] Mathiesen C, Caesar K, Akgören N, Lauritzen M (1998). Modification of activity-dependent increases of cerebral blood flow by excitatory synaptic activity and spikes in rat cerebellar cortex. *The Journal of Physiology*.

[13] Palacios JM, Kuhar MJ, Rapoport SI, London ED (1982). Effects of *γ*-aminobutyric acid agonist and antagonist drugs on local cerebral glucose utilization. *Journal of Neuroscience*.

[14] Kelly PAT, Ford I, McCulloch J (1986). The effect of diazepam upon local cerebral glucose use in the conscious rat. *Neuroscience*.

[15] Tagamets M-A, Horwitz B (2001). Interpreting PET and fMRI measures of functional neural activity: the effects of synaptic inhibition on cortical activation in human imaging studies. *Brain Research Bulletin*.

[16] Nunez P (1981). *Electric Fields of Brain*.

[17] Kilner JM, Mattout J, Henson R, Friston KJ (2005). Hemodynamic correlates of EEG: a heuristic. *NeuroImage*.

[18] Goldman RI, Stern JM, Engel J, Cohen MS (2002). Simultaneous EEG and fMRI of the alpha rhythm. *NeuroReport*.

[19] Martínez-Montes E, Valdés-Sosa PA, Miwakeichi F, Goldman RI, Cohen MS (2004). Concurrent EEG/fMRI analysis by multiway partial least squares. *NeuroImage*.

[20] Laufs H, Kleinschmidt A, Beyerle A (2003). EEG-correlated fMRI of human alpha activity. *NeuroImage*.

[21] Leopold DA, Murayama Y, Logothetis NK (2003). Very slow activity fluctuations in monkey visual cortex: implications for functional brain imaging. *Cerebral Cortex*.

[22] Müller-Putz GR, Zimmermann D, Graimann B, Nestinger K, Korisek G, Pfurtscheller G (2007). Event-related beta EEG-changes during passive and attempted foot movements in paraplegic patients. *Brain Research*.

[23] Müller-Putz GR, Linortner P, Winkler R, Korisek G, Pfurtscheller G BCI accuracy in healthy persons and SCI patients.

[24] Alkadhi H, Brugger P, Boendermaker SH (2005). What disconnection tells about motor imagery: evidence from paraplegic patients. *Cerebral Cortex*.

[25] Enzinger C, Ropele S, Fazekas F (2008). Brain motor system function in a patient with complete spinal cord injury following extensive brain-computer interface training. *Experimental Brain Research*.

[26] Formaggio E, Storti SF, Avesani M (2008). EEG and fMRI coregistration to investigate the cortical oscillatory activities during finger movement. *Brain Topography*.

[27] Dale AM, Liu AK, Fischl BR (2000). Dynamic statistical parametric mapping: combining fMRI and MEG for high-resolution imaging of cortical activity. *Neuron*.

[28] He B, Hori J, Babiloni F, Akay M (2006). EEG inverse problems. *Wiley Encyclopedia in Biomedical Engineering*.

[29] Im C-H, Liu Z, Zhang N, Chen W, He B (2006). Functional cortical source imaging from simultaneously recorded ERP and fMRI. *Journal of Neuroscience Methods*.

[30] Liu Z, He B (2008). fMRI-EEG integrated cortical source imaging by use of time-variant spatial constraints. *NeuroImage*.

[31] Babiloni F, Babiloni C, Carducci F (2003). Multimodal integration of high-resolution EEG and functional magnetic resonance imaging data: a simulation study. *NeuroImage*.

[32] Babiloni F, Cincotti F, Babiloni C (2005). Estimation of the cortical functional connectivity with the multimodal integration of high-resolution EEG and fMRI data by directed transfer function. *NeuroImage*.

[33] Urbano A, Babiloni C, Onorati P (1998). Responses of human primary sensorimotor and supplementary motor areas to internally triggered unilateral and simultaneous bilateral one-digit movements. A high-resolution EEG study. *European Journal of Neuroscience*.

[34] Babiloni F, Babiloni C, Locche L, Cincotti F, Rossini PM, Carducci F (2000). High-resolution electro-encephalogram: source estimates of Laplacian-transformed somatosensory-evoked potentials using a realistic subject head model constructed from magnetic resonance images. *Medical & Biological Engineering & Computing*.

[35] Kaminski MJ, Blinowska KJ (1991). A new method of the description of the information flow in the brain structures. *Biological Cybernetics*.

[36] Granger CWJ (1969). Investigating causal relations by econometric models and cross-spectral methods. *Econometrica*.

[37] Astolfi L, Cincotti F, Mattia D (2004). Estimation of the effective and functional human cortical connectivity with structural equation modeling and directed transfer function applied to high-resolution EEG. *Magnetic Resonance Imaging*.

[38] Astolfi L, Cincotti F, Mattia D (2005). Assessing cortical functional connectivity by linear inverse estimation and directed transfer function: simulations and application to real data. *Clinical Neurophysiology*.

[39] Astolfi L, Cincotti F, Mattia D (2007). Comparison of different cortical connectivity estimators for high-resolution EEG recordings. *Human Brain Mapping*.

[40] Astolfi L, De Vico Fallani F, Cincotti F (2007). Imaging functional brain connectivity patterns from high-resolution EEG and fMRI via graph theory. *Psychophysiology*.

[41] Astolfi L, De Vico Fallani F, Cincotti F Estimation of effective and functional cortical connectivity from neuroelectric and hemodynamic recordings.

[42] Salek-Haddadi A, Merschhemke M, Lemieux L, Fish DR (2002). Simultaneous EEG-correlated ictal fMRI. *NeuroImage*.

[43] Salek-Haddadi A, Lemieux L, Merschhemke M, Friston KJ, Duncan JS, Fish DR (2003). Functional magnetic resonance imaging of human absence seizures. *Annals of Neurology*.

[44] Kobayashi E, Hawco CS, Grova C, Dubeau F, Gotman J (2006). Widespread and intense BOLD changes during brief focal electrographic seizures. *Neurology*.

[45] Geselowitz DB (2004). Introduction to “some laws concerning the distribution of electric currents in volume conductors with applications to experiments on animal electricity”. *Proceedings of the IEEE*.

[46] Ives JR, Warach S, Schmitt F, Edelman RR, Schomer DL (1993). Monitoring the patient's EEG during echo planar MRI. *Electroencephalography and Clinical Neurophysiology*.

[47] Huang-Hellinger FR, Breiter HC, McCormack G (1995). Simultaneous functional magnetic resonance imaging and electrophysiological recording. *Human Brain Mapping*.

[48] Warach S, Ives JR, Schlaug G (1996). EEG-triggered echo-planar functional MRI in epilepsy. *Neurology*.

[49] Seeck M, Lazeyras F, Michel CM (1998). Non-invasive epileptic focus localization using EEG-triggered functional MRI and electromagnetic tomography. *Electroencephalography and Clinical Neurophysiology*.

[50] Symms MR, Allen PJ, Woermann FG (1999). Reproducible localization of interictal epileptiform discharges using eeg-triggered fMRI. *Physics in Medicine and Biology*.

[51] Krakow K, Woermann FG, Symms MR (1999). EEG-triggered functional MRI of interictal epileptiform activity in patients with partial seizures. *Brain*.

[52] Krakow K, Lemieux L, Messina D (2001). Spatio-temporal imaging of focal interictal epileptiform activity using EEG-triggered functional MRI. *Epileptic Disorders*.

[53] Lazeyras F, Blanke O, Perrig S (2000). EEG-triggered functional MRI in patients with pharmacoresistant epilepsy. *Journal of Magnetic Resonance Imaging*.

[54] Archer JS, Briellman RS, Abbott DF, Syngeniotis A, Wellard RM, Jackson GD (2003). Benign epilepsy with centro-temporal spikes: spike triggered fMRI shows somato-sensory cortex activity. *Epilepsia*.

[55] Lemieux L, Salek-Haddadi A, Josephs O (2001). Event-related fMRI with simultaneous and continuous EEG: description of the method and initial case report. *NeuroImage*.

[56] Salek-Haddadi A, Diehl B, Hamandi K (2006). Hemodynamic correlates of epileptiform discharges: an EEG-fMRI study of 63 patients with focal epilepsy. *Brain Research*.

[57] Liston AD, De Munck JC, Hamandi K (2006). Analysis of EEG-fMRI data in focal epilepsy based on automated spike classification and Signal Space Projection. *NeuroImage*.

[58] Luck SJ (2005). *An Introduction to the Event-Related Potential Technique*.

[59] Ives JR, Warach S, Schmitt F, Edelman RR, Schomer DL (1993). Monitoring the patient's EEG during echo planar MRI. *Electroencephalography and Clinical Neurophysiology*.

[60] Lemieux L, Allen PJ, Franconi F, Symms MR, Fish DR (1997). Recording of EEG during fMRI experiments: patient safety. *Magnetic Resonance in Medicine*.

[61] Bonmassar G, Anami K, Ives J, Belliveau JW (1999). Visual evoked potential (VEP) measured by simultaneous 64-channel EEG and 3T fMRI. *NeuroReport*.

[62] Mullinger K, Debener S, Coxon R, Bowtell R (2008). Effects of simultaneous EEG recording on MRI data quality at 1.5, 3 and 7 tesla. *International Journal of Psychophysiology*.

[63] Bénar C-G, Schön D, Grimault S (2007). Single-trial analysis of oddball event-related potentials in simultaneous EEG-fMRI. *Human Brain Mapping*.

[64] Wesensten NJ, Badia P, Harsh J (1990). Time of day, repeated testing, and interblock interval effects on P300 amplitude. *Physiology and Behavior*.

[65] Novitski N, Anourova I, Martinkauppi S, Aronen HJ, Näätänen R, Carlson S (2003). Effects of noise from functional magnetic resonance imaging on auditory event-related potentials in working memory task. *NeuroImage*.

[66] Eichele T, Specht K, Moosmann M (2005). Assessing the spatiotemporal evolution of neuronal activation with single-trial event-related potentials and functional MRI. *Proceedings of the National Academy of Sciences of the United States of America*.

[67] Näätänen R (1992). *Attention and Brain Function*.

[68] Debener S, Ullsperger M, Siegel M, Fiehler K, von Cramon DY, Engel AK (2005). Trial-by-trial coupling of concurrent electroencephalogram and functional magnetic resonance imaging identifies the dynamics of performance monitoring. *Journal of Neuroscience*.

[69] Debener S, Ullsperger M, Siegel M, Engel AK (2006). Single-trial EEG-fMRI reveals the dynamics of cognitive function. *Trends in Cognitive Sciences*.

[70] Marzetti L, Mantini D, Cugini S, Romani GL, Del Gratta C High-resolution spatio-temporal neuronal activation in the visual oddball task: a simultaneous EEG/fMRI study.

[71] Eichele T, Calhoun VD, Moosmann M (2008). Unmixing concurrent EEG-fMRI with parallel independent component analysis. *International Journal of Psychophysiology*.

[72] Moosmann M, Eichele T, Nordby H, Hugdahl K, Calhoun VD (2008). Joint independent component analysis for simultaneous EEG-fMRI: principle and simulation. *International Journal of Psychophysiology*.

